# Senescence Alterations in Pulmonary Hypertension

**DOI:** 10.3390/cells10123456

**Published:** 2021-12-08

**Authors:** Inés Roger, Javier Milara, Nada Belhadj, Julio Cortijo

**Affiliations:** 1 Centro de Investigación en Red Enfermedades Respiratorias CIBERES, Health Institute Carlos III, 28029 Valencia, Spain; julio.cortijo@uv.es; 2 Department of Pharmacology, Faculty of Medicine, University of Valencia, 46010 Valencia, Spain; belhadj@alumni.uv.es; 3 Pharmacy Unit, University General Hospital Consortium of Valencia, 46014 Valencia, Spain; 4 Research and Teaching Unit, University General Hospital Consortium, 46014 Valencia, Spain

**Keywords:** senescence, pulmonary hypertension, SASP, senolytics

## Abstract

Cellular senescence is the arrest of normal cell division and is commonly associated with aging. The interest in the role of cellular senescence in lung diseases derives from the observation of markers of senescence in chronic obstructive pulmonary disease (COPD), pulmonary fibrosis (IPF), and pulmonary hypertension (PH). Accumulation of senescent cells and the senescence-associated secretory phenotype in the lung of aged patients may lead to mild persistent inflammation, which results in tissue damage. Oxidative stress due to environmental exposures such as cigarette smoke also promotes cellular senescence, together with additional forms of cellular stress such as mitochondrial dysfunction and endoplasmic reticulum stress. Growing recent evidence indicate that senescent cell phenotypes are observed in pulmonary artery smooth muscle cells and endothelial cells of patients with PH, contributing to pulmonary artery remodeling and PH development. In this review, we analyze the role of different senescence cell phenotypes contributing to the pulmonary artery remodeling process in different PH clinical entities. Different molecular pathway activation and cellular functions derived from senescence activation will be analyzed and discussed as promising targets to develop future senotherapies as promising treatments to attenuate pulmonary artery remodeling in PH.

## 1. Introduction

### 1.1. Definition

Cellular senescence was first defined by Hayflick and Moorhead (1961) who reported that normal human fibroblasts have a finite replicative lifespan [1]. Currently we refer to cellular senescence as the arrest of normal cell division in response to a variety of cellular stresses or DNA damage along with proinflammatory response, mitochondrial dysfunction, and telomere shortening [2]. In this process, cells lose their ability to proliferate, accompanied in most cases by the expression of an inflammatory phenotype called the senescent-associated secretory phenotype (SASP), which is capable of influencing the microenvironment [3,4].

**Figure 1:**
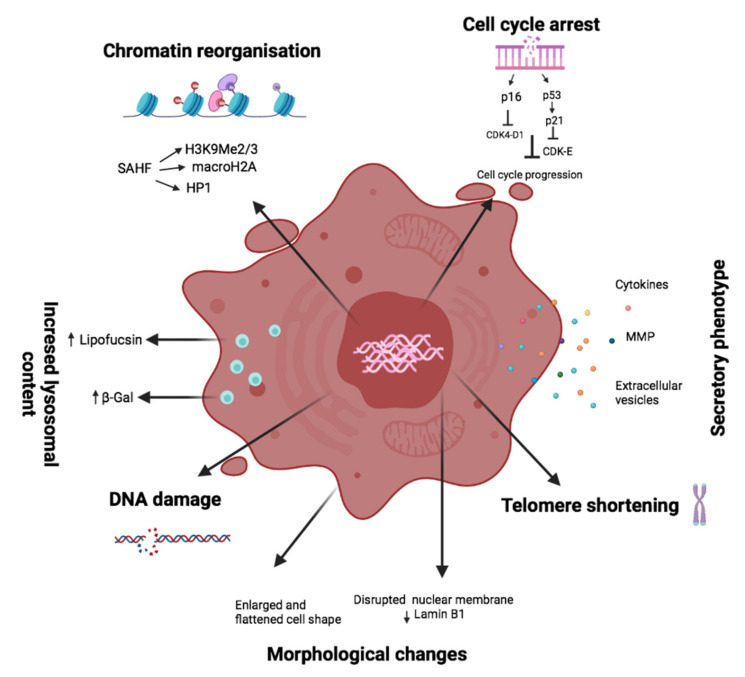
Hallmarks of cellular senescence. A large number of cellular processes are involved in the development of senescence. These include: morphological changes and macromolecular damage; increased lysosomal compartment, which is characterized by the overexpression of β-Gal; chromatin reorganization, which includes senescence-associated heterochromatin foci (SAHF); irreversible cell cycle arrest, driven by the action of p16 and p21/p53 axes, depending on the senescence driver and the implementation of a secretory phenotype, known as senescent-associated secretory phenotype (SASP) and characterized by the release of matrix metalloproteinases (MMP), cytokines and extracellular vesicles. Although these markers are strongly associated with a senescent phenotype, they are not exclusive or essential for the development of the program (with the exception of cell cycle arrest).

Cellular senescence has since been identified as a response to numerous stressors, including exposure to genotoxic agents, nutrient derivation, hypoxia, mitochondrial dysfunction, and oncogene activation [5]. The senescence phenotype is often characterized by irreversible cell cycle arrest; increased secretion of pro-inflammatory and tissue-remodeling factors; structural aberrations, from enlarged and more flattened morphology; macromolecular damage; chromatin reorganization; altered metabolic rates; accumulation of lysosomes content; and resistance to apoptosis [5,6,7] (Figure 1).

Since its discovery two decades ago, many studies have been performed to determine the functions of cellular senescence in human diseases. These studies have revealed that cellular senescence is involved in an increasing number of disease processes in many different cell types and tissues. Cell senescence is associated with a variety of diseases, such as diabetes, osteoarthritis, non-alcoholic fatty liver disease, tumorigenesis, pathogenic infection, idiopathic pulmonary fibrosis (IPF), and pulmonary hypertensions. Depending on the pathology, cellular senescence has different affects: deleterious or beneficial. 

### 1.2. Types of Senescence

**Figure 2:**
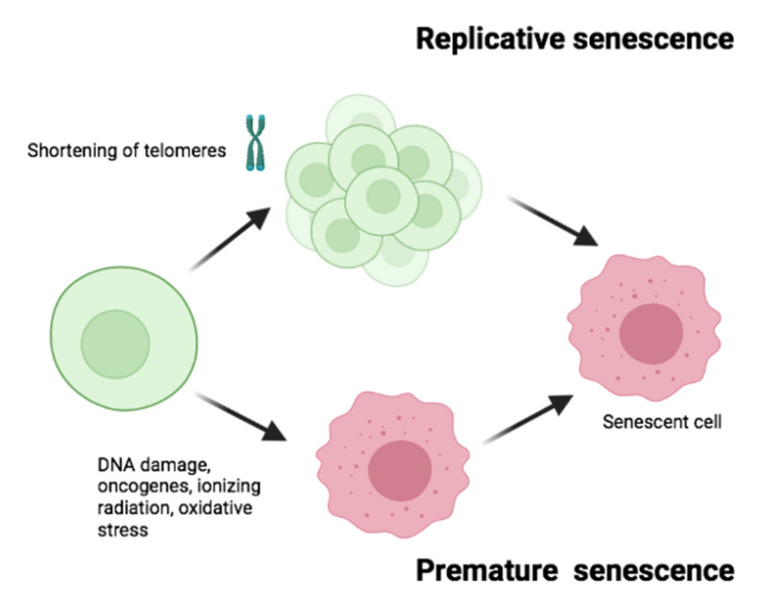
Types of senescence. Cellular senescence may be triggered by two different mechanisms: Replicative senescence and premature senescence. Replicative senescence refers to the decrease in proliferation due to shortening of telomeres as a consequence of multiple cell division. While premature senescence occurs in response to various stress stimuli, such as DNA damage, oncogenes, ionizing radiation, or oxidative stress.

In vitro senescence can be induced by different stimuli [8]. Depending on the stimulus, the type of senescence may be different. Whether all these “types of senescence” actually occur in vivo is not yet known. We can point out and differentiate mainly two types of senescence triggered by two different mechanisms: replicative senescence or premature senescence (Figure 2). Replicative senescence refers to the decrease in proliferation potentially observed after multiple cell divisions, leading to shortening of telomeres that ultimately results in total arrest [9]. It has also been hypothesized that this is a phenomenon that occurs mainly in vitro [10]. In contrast, premature senescence may occur in response to various stress stimuli, such as oxidative stress, oncogenes or ionizing radiations [11]. Below we will briefly describe different types of premature senescence.

DNA damage-induced senescence occurs when irreparable damage in DNA induces either senescence or apoptosis, depending on the magnitude of the damage. In vitro, multiple DNA-damaging agents are used to induce this type of senescence, including radiation (UV or ionizing) or multiple drugs such as bleomycin or doxorubicin [8].

Oncogene-induced senescence (OIS) is another type of senescence within premature senescence. Overexpression of some oncogenes, such as Ras, Raf, Akt, E2F1/3, and Cyclin E, or the inactivation of tumor suppressors such as PTEN can also induce premature senescence [9].

Furthermore, either oxidizing products of the cell metabolism or known oxidative agents (e.g., H_2_O_2_ or tert-butylhydroperoxide) can cause senescence, known as oxidative stress-induced senescence [12,13]. Although oxidizing agents exert their effect partly through DNA damage, other cellular components and processes are also affected. 

Finally, we highlight two more types of premature senescence. The mitochondrial dysfunction-associated senescence (MiDAS) was recently reported. Induction of mitochondrial dysfunction also leads to senescence. The phenotype, particularly the SASP, seems to be characteristic of this type of senescence [14] and senescence-associated epigenetics, which is characterized by changes in gene expression and induced by inhibitors of DNA methylases (e.g., 5-aza-2′-deoxycytidine) or histone deacetylases (e.g., sodium butyrate) [5,15]. 

### 1.3. Senescence Markers

The phenotype associated with cellular senescence is highly variable and heterogeneous. Marker specificity varies depending on cell type, tissue, organismal developmental stage, species, and other factors. The diversity of phenotype is reflected by the lack of specific markers and by the necessity to measure multiple facultative senescence-associated markers [6,16]. The lack of universal or program-specific markers is a major limitation for the identification and targeting of senescent cells in vitro and in vivo.

The best marker, however, should address both in vivo and in vitro conditions, apply to various tissues and species, and show consistency and overlapping with other reliable markers. Telomere shortening meets these requirements and shares similar trends with a majority of various other senescence-associated cellular markers. However, there are other senescent markers that we describe below (Table 1).

The lysosomal hydrolase β-galactosidase remains one of the most popular markers of cellular senescence, particularly of replicative senescence [17]. Enzyme activity of β-galactosidase in normal cells can be detected at pH 4, however, the activity of the enzyme in a senescent cell is observed at pH6 [18,19].

Senescence is also associated with chromatin morphological changes. Heterochromatin foci are facultative heterochromatin domains associated with irreversible cell cycle arrest and accumulate in senescent cells [20]. Structurally, senescence-associated heterochromatin foci (SAHF) contain silent heterochromatin domains consisting of a histone H3 di- or tri-methylated lysine 9 (H3K9Me2/3), a variant of histone H2A (macroH2A), and heterochromatin protein 1 (HP1) proteins [21,22]. SAHF is not a universal marker of cell aging but is a specific tissue and species senescent marker [23]. SAHF can be induced in senescent cells by oncogenes, toxic agents, or telomere shortening [24,25].

Ser-139 at the C-terminus of the H2AX molecule is phosphorylated to produce γH2AX [26]. Histone γH2AX is the most sensitive marker for double-stranded DNA breaks (DSB) and telomere shortening [27]. The number of γH2AX foci increase in damaged and senescent cells in most tissues and species, both in vivo and in vitro [28,29,30]. However, γH2AX may not be a good marker of cellular senescence due to a lack of specificity since the amount of γH2AX can be reduced following DSB repair. Another traditional marker of the DNA repair processes is p53-binding protein 1 (53BP1), which is phosphorylated in response to DNA damage and is an indicator of senescence [16]. 

Lamin B1 is another biomarker of senescence that is lost when cells are induced to senescence due to changes in organelle structures [31].

Finally, we would like to mention markers involved in cell cycle arrest and SASP. Cell cycle arrest was driven by the action and cooperation of several proteins implicated in the p16/Rb and p21/p53 axes, depending on the senescence driver [7]. The SASP includes several families of soluble and insoluble factors. These factors can be globally divided into the following major categories: soluble signaling factors (interleukins, chemokines and grown factors), secreted proteases, ROS, and secreted insoluble proteins/extracellular matrix (ECM) [32].

### 1.4. Vessels and Senescence

Aging is a risk factor for the vast majority of cardiovascular diseases, and in turn, many cardiovascular diseases are associated with premature vascular aging and vascular cell senescence [33]. Vessel aging leads to intimal and medial thickening (vascular remodeling), loss of arterial elasticity, increased collagen deposition, fracture of the elastin lamellae, and endothelial dysfunction. Vascular senescence cells can impair vascular functions and contribute to vascular diseases. In addition, aged vessels show elevated expression of several proinflammatory molecules, resulting in persistent vascular inflammation [33].

Cellular senescence is recognized as a crucial contributor to the pathobiology of vascular diseases, such as stroke, coronary artery diseases, myocardial infarction, or pulmonary hypertension (PH). In atherosclerosis, especially, it is becoming evident that senescent cells play a deleterious role in disease development and progression [34]. Senescent vascular endothelial cells are predominately localized in the plaque of human atherosclerosis, but not in normal lesions, and result in endothelial dysfunction [35]. Senescent endothelial cells in these lesions produce less nitric oxide and prostacyclin, and both smooth muscle cells and endothelial cells develop a proinflammatory, profibrotic SASP. This leads to a dysfunctional vessel that is constricted and chronically inflamed, which favors plaque formation and leads to stiffening and systemic hypertension [36]. Senescent endothelial cells disrupt endothelial integrity and contribute to vascular aging. This may explain, in part, why the elderly population has an increased susceptibility for vascular diseases, such as coronary artery disease and PH [36]. The cellular and vascular abnormalities associated with senescence found in atherosclerosis are in many ways similar to those found in the pulmonary vasculature in PAH [37].

## 2. Pulmonary Hypertension and Senescence

### 2.1. Definition and Classification

PH is defined as a group of diseases characterized by a progressive increase in pulmonary vascular resistance (PVR), which leads to right ventricular failure and premature death [38]. The term PH is defined as a mean pulmonary artery pressure (mPAP) greater than 25 mmHg, measured at rest according to the guidelines issued by the European Society of Cardiology (SEC) and by the European Respiratory Society (SER) [39].

The clinical classification of PH is intended to categorize multiple clinical conditions into five groups according to their clinical presentation, pathological manifestations, hemodynamic characteristics, and treatment strategies [39]. A comprehensive version of the clinical classification is presented in Table 2.

PH is a severe arteriopathy in which a characteristic form of neointimal vascular remodeling progressively occludes the pulmonary arteries. The vascular lesions observed in the various PH etiologies typically consist of dysfunctional pulmonary arterial endothelial cell (PAEC) and pulmonary arterial smooth muscle cells (PASMC) that have formed a neointima and media [37]. Recently, it has been established that senescence plays a significant role in this disease as an important contributor to vascular remodeling [41,42].

### 2.2. Expression and Distribution of Senescent Markers in Pulmonary Hypertension

Amongst the different groups of PH, the expression and distribution studies that analyze senescent markers focus mainly on PAH, and to a lesser extent, HFpEF and COPD-PH, and describe primarily p53, p21, p16, and Bcl2. Table 3 shows a summary of the studies that analyze senescent markers expression in animal models and cell types, and Table 4 shows a summary of the studies in patients with PH.

P53 and p21 are senescent markers that are involved in cycle cell arrest. P53 is overexpressed in PAECs from mice with hypoxia and rats with monocrotaline (MCT). However, its expression is downregulated in PASMCs from mice with hypoxia and rats with MCT [43,44]. The hypoxia and MCT-induced p53 decrease in PASMCs contributes to pulmonary vascular remodeling by stimulation of PASMCs proliferation and store-operated Ca^2+^ entry. Furthermore, the decreased p53 in PASMCs from these animal models was associated with a decrease of Bax, a pro-apoptotic protein, and an increase of Bcl-2, an anti-apoptotic protein, as well as HIF-1α levels. Moreover, PASMCs from iPAH patients exhibited significant decreases in the protein expression level of p53 and the Bax/Bcl-2 ratio compared with normal control PASMCs [43]. In contrast, PAECs from iPAH patients demonstrated significant elevation of p53 and Bax/Bcl-2 ratio [43,45]. Regarding the animal’s lung tissue with experimental PH, some studies found that the p53 protein expression was significantly lower in lungs from MCT-PH and hypoxic PH animal models, which might contribute to vascular remodeling with progression of PH [44,46,47]. Moreover, hypoxic pulmonary vascular remodeling is augmented in p53 knockout mice, and these effects may be associated with an increase of HIF-1a and loss of p21 expression in lung vessels [48].

Numerous studies have demonstrated that p21 is implicated in the vascular remodeling associated with senescence in PH. Hypoxic mice markedly increased lung p21 mRNA and protein levels in contrast to normoxia mice, and these increases are more pronounced in older mice [47,49]. Moreover, our group demonstrated that p21 mRNA and protein levels are increased in the experimental model of bleomycin-induced pulmonary fibrosis associated with PH, IL-11-treated mice, and MCT rats [50,51,52,53]. Finally, p21 is also upregulated in pulmonary vessels, mainly in plexiform lesions, from PAH, CDH-PH, and COPD-PH patients [41,54]

On the other hand, p16 also causes cell cycle arrest. P16-expressing cells have already been described in plexiform lesions and the intima of the vessels in end-stage iPAH and COPD-PH patients [49,54,55]. Similar results were observed in older hypoxic mice [41,49].

The overexpression of these markers implies an arrest of the cell cycle that manifests itself in a decrease in proliferation. However, senescent cells also upregulate specific senescent cell anti-apoptotic pathways such as Bcl2. Interestingly, high values of endothelial Bcl2 index are associated with PAH [56], especially portopulmonary PH, whereas lower values are associated with heart failure with preserved ejection fraction (HFpEF) [57]. Other studies revealed an elevated Bcl2 expression in pulmonary arterial walls in mice exposed to hypoxia [58]. Survivin is also highly expressed in luminal cells of severe lesions in PAH, while it is absent in controls [41]. 

SASP markers are also important to detect senescent cells. Various studies have demonstrated that MMP2 and IL-6 are upregulated in mice treated with MCT [41,59]. In addition, MMP2 and IL-6 levels are elevated in serum, urine, and lungs of patients with iPAH [60,61,62]. 

### 2.3. Signaling Pathways Involved in Cellular Senescence and in PH

**Figure 3:**
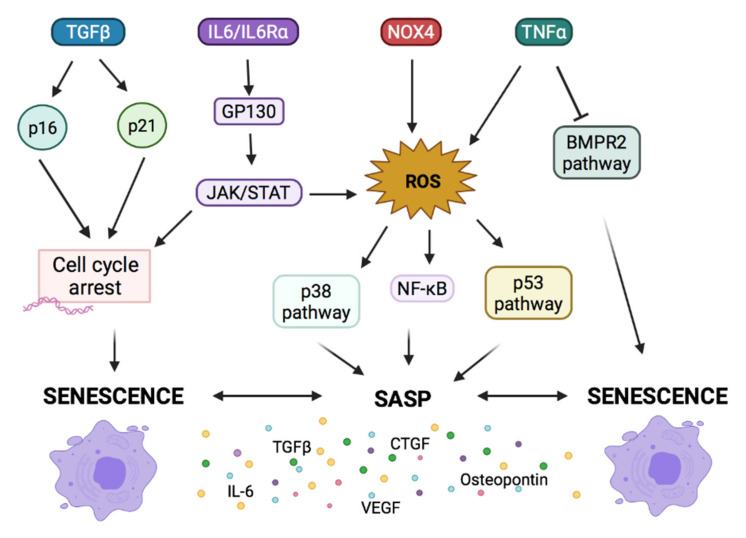
Mechanisms of cellular senescence. TGFβ activates the p21 and p16 pathway to stop the cell cycle, which induces senescence. The binding of IL-6 to its unique-receptor IL-6R triggers the homodimerization of GP130. This results in the phosphorylation of Janus kinases (JAK), which phosphorylate intracellular tyrosine residues that serve as docking sites for STAT3. JAK/STAT induces cell cycle arrest and causes the initial generation of reactive oxygen species (ROS), subsequent senescence, and senescence associated secretory phenotype (SASP) (expression of IL-1α, IL-1β, IL-6, CTGF, VEGF, TGFβ, and osteopontin). NOX4 and TNFα also induce ROS production. ROS can induce senescence and SASP through the p38MAPK/NF-κB/p53 pathway.

Elucidation of the mechanisms by which cell senescence may affect PH development and progression is of great clinical importance. In particular, it is relevant to identify the set of factors and mechanisms that lead to senescence and PH. Here, we review some factors and molecular mechanisms such as transforming growth factor- β (TGF-β), TNF-α, IL-6, nitric oxide, and osteopontin, which participate in PH and senescence (Figure 3).

#### 2.3.1. Growth Factors Imbalance Lead to Senescence and PH

Several growth factors are expressed in patients and animal models of PH. TGF-β expression is correlated with physiological alterations of the pulmonary vasculature and RV pressures [63] and contributes to the pathogenesis of PH [64,65]. Serum and lung tissue expression levels of TGF-β are elevated in patients with iPAH and PH associated to chronic lung disease (PH-CLD) [66]. The role of TGF-β in senescence may be both causal and consequential [67]. In cancer, it has been recognized that TGF-β is capable of generating senescence either by direct interaction with p16 and p21 [68] or by indirect action due to exacerbated ROS production and DNA damage by the suppression of adenine nucleotide translocate-2 (ANT2) [69,70]. On the other hand, the increase of TGF-β can also be a consequence of senescence because senescent cells release TGF-β as part of SASP [32]. The TGF-β component of the SASP is recognized as an important mediator of fibrosis in age-related diseases such as IPF [71].

On other hand, plasma connective tissue growth factor (CTGF) levels are higher in PAH associated with congenital heart disease (CHD) and in PAH patients [72], and it has been demonstrated that this growth factor is up-regulated in senescent cells and contributes, similar to TGF-β, to the induction of paracrine senescence [73,74].

Finally, high levels of vascular endothelial growth factor (VEGF) have been observed in PAH and PAH-CHD patients’ lung samples in plexiform lesions, however, adjacent arteries showed a faint expression [75,76,77]. High levels of VEGF are also a consequence of senescence, because senescent cells release these growth factors as a part of SASP. However, the exact secretome is dynamic and depends on cell type, microenvironment, cause of senescence, and the biological pathway by which the SASP is activated [78].

#### 2.3.2. Cytokines as Activators of Senescence in PH

Previous studies have proven that various inflammatory factors such as interleukin 6 (IL-6), TNF-α etc. are involved in pulmonary vascular remodeling in PH. However, the underlying mechanisms of these active substances promote senescence to remain completely elucidated.

Many previous studies have described the key role that interleukins of the IL-6 family play in the pathogenesis of PH, among which IL-6 and interleukin11 (IL-11) are the most relevant. The first demonstration of increased IL-6 dates to 1996 [61]; however, the involvement of IL-11 in PH was recently demonstrated by our group [52]. Both interleukins are overexpressed in the serum of patients with iPAH and PH-CLD, and high serum levels of IL-6 are correlated with worse prognoses [51,52,61,79,80]. The exact mechanism by which IL-11 promotes cell senescence has not yet been elucidated. However, our group has shown that stimulation with IL-11 promotes an increase in p21 expression and senescence-associated β-galactosidase (SA-β-Gal) activity both in vitro and in vivo [50,51,52]. As for IL-6, the information described is greater. It has been reported that it may be involved in the pathogenesis of senescence [81,82,83]. The stimulation with IL-6 + sIL-6Rα induced and increases SA-β-Gal activity, p53 protein expression, and ROS levels [81]. The combination of IL-6/sIL-6Rα complex and gp130 activates the JAK/STAT signal transduction pathway and transmits the signal from the cell membrane to nucleus, which is critical to cell cycle transition from G1 to S [84]. Moreover, IL-6/sIL-6Rα induces premature senescence through STAT3/p53/p21 pathway in vascular smooth muscle cells [85,86]. On the other hand, the increase of IL-6 can also be a consequence of senescence because senescent cells release IL-6 as part of SASP [86,87].

On the other hand, TNFα is recognized as a critical trigger for PAH [79] since transgenic mice that overexpress TNFα in the lung develop PAH [88] and TNFα expression is elevated in rats with HP induced with MCT [89]. Recent evidence indicates that increased TNFα directly promotes pulmonary vascular remodeling by reducing BMPR2 signaling [90]. Prolonged exposure of TNF-a is also known to induce senescence and SASP in endothelial cells and fibroblasts [91] via increased ROS and activation of the NF-kB and p38 MAPK pathway [92,93]. Increased ROS levels may be attributed to the weakened anti-oxidative response evidenced by the under-expression of Nrf2 gene [93].

#### 2.3.3. Imbalance in Mediators of Vascular Tone Leads to Senescence in PH

In patients with PH, the generation of vasodilatory mediators is reduced, and the generation of vasoconstrictor mediators is increased, which in turn increases the generation of reactive oxygen species and reactive nitrogen species, which play an important role in the development and/or progression of PH [94,95,96]. Moreover, senescent cells secrete an abnormal variety of molecules, including ROS, which modifies the cellular microenvironment, creating a vicious cycle of oxidative stress.

Regarding PH and endothelial nitric oxide synthase (eNOS), there are controversial results. Some reports have shown that in patients with PAH, CLD-PH and congenital heart disease associated with PH (CHD-PH), the expression of eNOS is reduced. Moreover, it has been reported that eNOS expression is correlated inversely with the severity of arterial remodeling [97,98,99]. However, other investigators have reported increased [100] or unaltered [101] eNOS immunostaining in human lungs with PH. In plexiform lesions in PAH, a high expression of eNOS has also been reported [102,103]. However, in senescent endothelial cells, the activity of eNOS and the production of NO are diminished [104]. Furthermore, a report showed that the effect of NO donors reduced cellular senescence and delayed age-dependent inhibition of telomerase activity, thus the production of NO diminished and had an adverse effect [105].

Although several enzymes produce ROS, the most important is NADPH oxidase, which plays a key role in the remodeling and vasoconstrictive aspects of PH [106]. Seven enzyme subtypes of Nox have been identified in a wide range of cell types, but only Nox1, Nox2, Nox4, and Nox5 are found in the pulmonary vasculature [107,108]. Nox4 is overexpressed in PASMCs and PAEC in PH and produces ROS [109,110]. The incensement of ROS can induce senescence through the p38MAPK/NF-κB/SASP pathway [111]. Nox4 could be epigenetically regulated. It is a direct target of miRNA-23b, miRNA-146a, or miRNA-25 that could upregulate the Nox4 expression and ROS levels, thereby leading to cellular senescence [109].

#### 2.3.4. Osteopontin and Senescence in PH

Osteopontin is an extracellular matrix protein released by senescent PASMC. The expression of this protein is increased in pulmonary arteries of patients with COPD-PH and iPAH [49,112], as well as in patient with advanced but not early PAH associated with CHD (PAH-CHD) [113]. Moreover, a positive correlation between osteopontin and age has been observed [49,112]. Senescent PASMC were recently shown to secrete a high concentration of osteopontin as a key component of their SASP and its release stimulated the migration and proliferation of target cells [49]. Osteopontin triggers its response through the autocrine or paracrine pathway, that is, through the release of components of the extracellular matrix or through the release of soluble factors [49]. However, the molecular pathway by which osteoponin induces senescence is not yet elucidated. 

## 3. Senotherapy in PH

Great progress has been made recently in understanding the molecular mechanisms involved in cellular senescence in PH. Although the complete reversal of normal aging has not been achieved, improvements have been achieved in the reversal of senescence in the vasculature of airways. Several new therapiy targets have been identified, leading to the development of senotherapies. 

We can differentiate two groups of senotherapy: (1) Senolytics drugs, which selectively destroy senescent cells, and (2) Senostatic drugs, which inhibit the pathways that lead to cellular senescence or suppress the release of SASP factors, which is to say, they reduce the pro-inflammatory phenotype of senescent cells. Gene therapies and immunotherapies are being explored as well, however, currently there are no data on PH [114].

Between senolytics and SASP suppression, senolytics hold the most therapeutic benefit because permanent removal of the senescent cell leads to a durable abolishment of deleterious SASP components [114]. Due to the close relationship observed between PH and senescence, senotherapy may offer a new approach to treatment. Table 5 provides an overview of the various classes of drugs that have been developed as senotherapies. Some of these drugs have already been evaluated in the context of PH.

### 3.1. Senolytic Therapy

Senolytics are drugs that induce apoptosis in senescent cells while having little or no effects on proliferating cells [115]. Despite arising from different tissues, groups of diseases, and cell types, a senescent cell may share similar biochemistry. This allows the reuse of therapeutic strategies across different groups of PH in which senescence is causal. Moreover, senescent cells mainly have negative effects on disease, reducing the possibility of senolysis-related side effects [114].

Dasatinib can serve as a senolytic, killing senescent cells by transiently suppressing senescence-associated anti-apoptotic pathways that are highly activated in senescence cells and protect these cells from surrounding the pro-apoptotic microenvironment [71,116]. However, incident cases of precapillary PH have been reported in CML patients treated with dasatinib. At diagnosis, patients had moderate to severe precapillary PH with functional and hemodynamic impairment. Generally, improvements were seen after dasatinib discontinuation (Clinicaltrials.gov identifier NCT01805843) [117]. In this same line, dasatinib causes pulmonary vascular damage, induction of ER stress, and mitochondrial ROS production, which leads to increased susceptibility to PH development in MCT-PH and hypoxic PH animal models [118].

There is no more data from clinical trials of senolytics therapies, but pre-clinical trials have been carried out in PH animal models. Navitoclax administration reduced SASP-dependent interstitial immune cell elevation in the vasculature, vessel remodeling, and consequent hemodynamic manifestation of PH in hypoxic mice and hypoxic IL-6 transgenic mice [119]. Hsp90 inhibitors have been identified as a novel class of senolytics because Hsp90 prevents apoptosis of senescent cells; therefore, its inhibition induces apoptosis [120]. Gamitrinib, a mitochondria-targeted Hsp90 inhibitor, improves MCT-induced PH in rats, reducing mPAP, RVSP, and vascular remodeling of distal pulmonary arteries [121]. Moreover, other studies have shown 17-AAG, Hsp90 inhibitor, improves the progress of PH demonstrated by lower pulmonary arterial pressure, absence of right ventricular hypertrophy, and improved pulmonary vascular remodeling in MCT rats [122]. However, none of the trials has assessed the drugs in direct relation to senescence or SASP in pulmonary hypertension.

Although there are practically no data from clinical trials of senolytics in patients with PH, the potential of these drugs in the treatment of PH is great. Thus, several biotechnology and pharmaceutical companies are searching for senolytic effective therapies to inhibit the development of senescence by targeting the signaling pathways that produce it in each disease [123].

### 3.2. Senostatic Therapy

**Figure 4:**
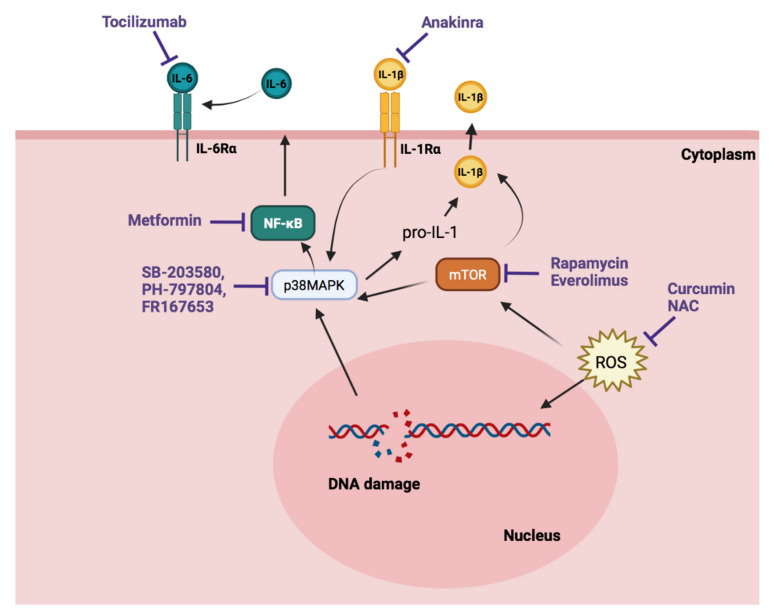
Pathways and inhibitors of cellular senescence. MAPK: mitogen-activated protein kinase; mTOR: mammalian target of rapamycin; NAC: N-acetyl cysteine; NF-κB: nuclear factor-kappa B; ROS: reactive oxygen species.

An additional approach to senotherapy would be to disrupt the SASP, which contains pro-inflammatory cytokines, growth factors, matrix metalloproteinases, and other bioactive molecules that are implicated in the disease (Figure 4). Even though senostatic drugs that target different molecular pathways involved in SASP have been described, most of these drugs have not been tested in direct relation to senescence in pulmonary hypertension. Therefore, a more in-depth investigation is needed to find out if senescent markers are affected by the use of these drugs.

Inhibitors of the PI3K–mTOR pathway may extend lifespan. The inhibition of mTOR by rapamycin has been shown to reverse or regress PH in several animal models [124,125,126,127]. Moreover, one clinical trial is being conducted with rapamycin for patients with severe PAH (Clinicaltrials.gov identifier NCT02587325). Ten patients with PAH or chronic thromboembolic PH have been included in a prospective open-label pilot study with everolimus. In two patients, study medication was stopped prematurely because of an adverse event. The remaining eight patients exhibited a significant decrease in PVR and increase in 6MWD after 6 months of treatment with everolimus [128]. Pre-clinical studies have also been carried out with everolimus, but all of them study the effects of everolimus in combination with sildenafil, not as a single therapy [129,130].

Regarding NF-κB inhibitors, Metformin is the most studied. Several pre-clinical studies have been performed with metformin in PH. These studies identified metformin as an effective therapeutic agent in well-established models of severe PH, MCT-induced PH, and hypoxia-induced PH rats. Metformin improved hemodynamic parameters and right ventricle hypertrophy. Moreover, this drug has an efficient anti-remodeling effect on pulmonary vasculature, improved endothelial function, decreased pulmonary artery contractility, and inhibited pulmonary artery proliferation [131,132,133]. On the other hand, five clinical trials are being carried out with metformin in PH. In HFpEF-PH, a prospective phase II clinical trial is evaluating the therapeutic efficacy of metformin (Clinicaltrials.gov identifier NCT03629340), which will evaluate exercise hemodynamics, functional capacity, and skeletal muscle signaling [134]. Moreover, three clinical trials (Clinicaltrials.gov identifier NCT03617458, NCT01352026 and NCT01884051) are looking at the role of metformin in PAH. In the study identified as NCT01884051, metformin therapy appeared to be safe and well-tolerated in PAH patients and was associated with improved right ventricle function [135]. Finally, patients with PAH-CHD have been randomized to receive bosetan with or without metformin, and the combination therapy provides improvements in important outcomes such as exercise capacity and pulmonary hemodynamics, compared with bosetan alone [136].

Another pathway investigated is apoptosis signal-regulating kinase 1 (ASK1). Selonsertib, a ASK1 inhibitor, has been evaluated in a double-blind study in PAH patients (Clinicaltrials.gov identifier NCT02234141). In this study, selonsertib had no significant effect on pulmonary vascular resistance in patients with PAH who were on stable therapy compared with placebo [137]. This study was performed because of accumulating evidence in pre-clinical studies that indicate that ASK1 inhibition might represent a novel therapeutic strategy for PAH since studies have shown that selonsertib oral administration to monocrotaline and Sugen/hypoxia rats reduced pulmonary arterial pressure and reduced RV hypertrophy. The effect of this drug in pre-clinical studies is related to the reduction of p38 and JNK phosphorylation [138].

Expression of p38 MAPK increased in both hypoxic and MCT rats models of PH [139]. Furthermore, its role in senescence has been described in detail. For this reason, different pre-clinical studies have been carried out with different inhibitors. The pretreatment with SB203580, a specific p38 MAPK inhibitor, caused a complete reversal of the impaired endothelium-dependent relaxation, secondary to both acute and chronic hypoxia [140]. Moreover, other studies demonstrated that right ventricle systolic pressure and superoxide anion production was lower in animals treated with p38 MAPK inhibitor than in the hypoxic group [139,141]. PH-797804 is another p38 MAPK inhibitor but this one is more specific and tolerated in humans [139]. Chronic hypoxic and MCT-induced PH was reversed with a PH-797804. Both SB203580 and PH-797804 reduced the production of tissue and circulating IL-6 in vivo [139]. FR167653 is another specific inhibitor of p38 mitogen, and the results in pre-clinical studies have shown similar outcomes. FR167653 attenuates vascular proliferation and reduces mean pulmonary artery pressure in MCT-induced PH in rats [142]. Despite any clinical trial that has been performed with these inhibitors in PH, PH-797804 has been evaluated in COPD patients and has demonstrated improvements over placebo in lung function parameters and dyspnea in patients with moderate to severe COPD [143]. Given that on many occasions, some patients with advanced COPD developed PH, this treatment could be the first step into a new therapy in COPD-PH.

Another way to inhibit cellular senescence is by suppressing oxidative stress. Natural chemicals with antioxidant potential can suppress oxidative stress-induced senescence. For example, curcumin, a natural phenol with antioxidant and anti-inflammatory activities, can attenuate hydrogen peroxide-induced premature endothelial cell senescence by activating Sirt1 [144]. In MCT-induced PH curcumin, administration was associated with reduced right ventricular wall thickness and a decreased right ventricle weight/body weight ratio [145]. Moreover, some molecules can delay senescence by directly suppressing ROS production. The antioxidant EUK-134 has been assessed in MCT rats and the treatment attenuated cardiomyocyte hypertrophy and the development of PAH [146]. N-acetylcysteine (NAC) is another antioxidant therapy employed in pre-clinical studies. The results demonstrated that (NAC) reduced the right ventricular hypertrophy index, mean pulmonary artery pressure, PVR, and pulmonary inflammation [147]. Currently, clinical trials are also evaluating the use of NAC in patients with chronic thromboembolic PH (Clinicaltrials.gov identifier NCT04081012).

Finally, we would like to mention the therapies that are aimed at inhibiting different interleukins that participate in the development of senescence such as IL1 α/β, TNFα, or IL-6. There is substantial evidence in the literature that describes an elevated activity of the cytokine IL-6 in the development of PH. Tocilizumab, a IL-6Rα inhibitor, is effective in animal models of PH, and currently a phase II clinical trial is being carried out in patients with group 1 PAH (Clinicaltrials.gov identifier NCT02676947). In summary, treatment with this inhibitor is feasible in PAH but it has not demonstrated any significant effects on hemodynamics or exploratory secondary endpoints in iPAH. However, a potential improvement was noted in a subgroup of patients with CTD-PAH [148].

Serum levels of IL-1β, which also promotes IL-6 synthesis, are also augmented in PH patients [79]. Anakinra is a recombinant of the IL-1β receptor α antagonist and is approved for the treatment of rheumatoid arthritis [149]. Experimental animal data suggests that anakinra protects against the development of PAH [150,151]. Taking these results, an open-label study of iPAH patients was performed. Anakinra was shown to have a safe profile (Clinicaltrials.gov identifier NCT03057028). This study also indicates that the drug in PAH has a trend reduction of IL-6 and a substantial improvement in symptoms, but a longer and larger study is necessary [152].

Finally, the administration of Etanercept, a TNFα inhibitor, was shown to improve the disease progression and re-establish the normal BMP/NOTCH pathway in MCT rats [153,154] and endotoxin-induced PH in pigs [155]. These results justify the need for anti-TNFα approaches in the treatment of PAH, but no study is currently investigating the safety and efficacy effects in PH patients.

**Table 5:** Overview of the various classes of drugs that have been developed as senotherapies. Some of these drugs have already been evaluated in the context of PH.

Group	Drug	Mechanism of Action	Groups of PH	Data of Trials in PH
Senolytics	Dasatinib	Inhibits BCR/ABL kinase. Targets multiple antiapoptotic pathways.	Clinical study	Incident cases of precapillary PH have been reported in patients who have chronic myelogenous leukemia [117] AM.
			Pre-clinical PH models	Dasatinib exaggerates the response to MCT and hypoxia [118] AM.
	Navitoclax (ABT263)	Bcl2 inhibitor.	Pre-clinical PH models	ABT263 reduced SASP elevation, vessel remodeling, and consequent hemodynamic manifestation in hypoxic mice and hypoxic IL-6 transgenic mice [119] AM.
	FOXO4-DRI	Stimulates p53-mediated apoptosis of senescent cells.		No data in PH.
	HSP90 inhibitors *	Induces apoptosis in senescent cells.	Pre-clinical PH models	Hsp90 inhibitors reduce mPAP, RVSP, and vascular remodeling and right ventricular hypertrophy in MCT rats [121,122] AM.
	UBX0101	Stimulates p53-mediated apoptosis of senescent cells.		No data in PH.
Senostatic	Rapamycin *	mTOR inhibitor	Clinical study: Severe PAH	Phase I (NCT02587325)
			Pre-clinical PH models	Rapamycin attenuates pulmonary vascular remodeling and right ventricular hypertrophy in MCT rats and hypoxic mice [122,123,124,125].
	Everolimus *		Clinical study: PAH and chronic thromboembolic PH	Everolimus decreased iPVR and increases 6MWD [128] AM.
	Metformin *	NF-κB inhibitor; suppression of the SASP.	Clinical study: CHD-PH	Metformin with bosetan provides improvements in important outcomes [136] AM.
			Clinical Study: PAH	Phase II (NCT03617458) Phase II (NCT01352026) Recruiting (NCT01884051).
			Clinical study: HFpEF-PH	Active, not recruiting (NCT03629340).
			Pre-clinical PH models	Metformin improved hemodynamic parameters and right ventricle hypertrophy in well-established models of severe PH.
	Selonsertib * (GS-444217)	ASK1 inhibition suppression of the SASP.	Pre-clinical PH models	GS-444217 reduced pulmonary arterial pressure and reduced RV hypertrophy in MCT and Sugen/hypoxia models [138] AM.
			Clinical study: PAH	Completed (NCT02234141). Selonsertib had no significant effect [137] AM.
	SB-203580 *	P38 MAPK inhibitor	Pre-clinical PH models	SB203580 caused decreased right ventricle systolic pressure, superoxide anion production, and the production of tissue and circulating IL-6 in hypoxic rats [139,140] AM.
	PH-797804 *		Pre-clinical PH models	Chronic hypoxic and MCT-induced PH was reversed with PH-797804. The production of tissue and circulating IL-6 was reduced too [137] AM.
	FR167653 *		Pre-clinical PH models	FR167653 attenuates vascular proliferation and reduces mean pulmonary artery pressure in MCT rats [142] AM.
	Curcumin	Antioxidant therapy	Pre-clinical PH models	Curcumin administration was associated with reduced right ventricular wall thickness and a decreased right ventricle weight/body weight ratio [145] AM.
	NAC		Pre-clinical PH models	NAC reduced right ventricular hypertrophy index, mean pulmonary artery pressure, PVR and pulmonary inflammation [147] AM.
			Clinical study: PAH	Recruiting (NCT04081012)
	Tocilizumab	IL-6Rα inhibitor	Clinical study: PAH	Phase II (NCT02676947)
	Anakinra	IL-1α receptor antagonist	Clinical study: PAH	Complete (NCT03057028)
			Pre-clinical PH models	Experimental animal data suggesting anakinra protects against development of PAH [150,151] AM.
	Etanercept	TNFα inhibitor	Pre-clinical PH models	Etanercept prevents and reverses MCT-PH in rats and endotoxin-PH in pigs5) [153,154,155].

ASK1: apoptosis signal-regulating kinase 1; HppEF-PH: heart failure with preserved ejection fraction associated to pulmonary hypertension; NAC: N-acetyl cysteine. * None of the trials assessed the drugs in direct relation to senescence or SASP in pulmonary hypertension.

## 4. Potential Application of Senolytics Therapies Used in Lung and Cardiac Disease and Perspectives on PH

An accumulation of senescence and increased inflammation, caused by the senescence-associated secretory phenotype, have been implicated in the etiology and progression of lung and cardiovascular diseases such as IPD, COPD, or atherosclerosis. Due to overwhelming evidence about the important contribution of cellular senescence to the pathogenesis of these diseases, specific targeting of senescent cells or of pathology-promoting SASP factors has been suggested as a potential therapeutic approach. However, due to the lack of a reliable marker able to detect senescence in vitro and in vivo, its precise impact of senescence in lung and cardiac disease is to a large extent still undetermined.

The administration of the senolytic navitoclax to 24 month-old mice reduces cardiomyocytes senescence, attenuates components of the cardiomyocytes SASP, reduces both interstitial fibrosis and cardiomyocytes hypertrophy, and reduces LV mass, all of which are characteristics of age-associated myocardial remodelling and HFpEF [156,157]. In the same line, recently two studies have provided evidence that senotherapies slow or prevent the progression of atherosclerosis [34,158]. Treatment of atherosclerotic *Ldlr*−/− mice with navitoclax, after senescence is established, reduces plaque burden and diminishes senescent cell numbers, plaque number, and the average plaque size. This is also associated with a reduction in factors implicated in plaque formation, such as IL-1α, MCP1, and TNFα [34]. The potential for senotherapies to abate atherosclerosis is also provided by the studies of Roos et al., using *ApoE−/−* mice, an alternative model of atherogenesis; treatment with dasatinib and querectin reduces senescence cell burden and plaque calcification but does not influence plaque size [158].

Regarding chronic lung disease, a combination of senolytics and senostatics have been evaluated as a new approach to COPD and IPF. Low-dose rapamycin reduced the number of senescence associated b-galactosidase-positive cells and increased proliferation of pulmonary artery endothelial cells from patients with COPD in vitro, with a reduction in SASP mediators [159]. In the same way, the inhibition of mTORC1 by rapamycin in a murine radiation lung injury model demonstrated decreased SASP cytokines and type II pneumocyte senescence, resulting in an overall decrease in pulmonary fibrosis [160].

Since cardiovascular and pulmonary diseases share pathophysiological characteristics with PH, targeting senescent cells in PAH with different senotherapies could be effective to attenuate pulmonary artery remodeling and hypertension. However, as described in detail in previous sections, there are no studies that describe in depth the direct relationship between senescent processes, senolytic drugs, and PAH.

## 5. The Pitfalls of Senescence in Pulmonary Hypertension

Although we described that many senescent markers such as IL-6, MMP2, and Bcl2, among others, are overexpressed in patients with PH, most of them participate in many other processes and are not specific to senescent processes. It could lead to misinterpretation of the data because the inhibition of some of them may have promoted improvements in the pathophysiology of PH and may not be due to the senolytic effects of the drug. For instance, navitoclax (ABT263), a Bcl2 inhibitor, has been used as a senolytic drug and has been shown to reduce SASP elevation, vessel remodeling, and consequent hemodynamic manifestation in hypoxic mice and hypoxic IL-6 transgenic mice [119]. However, maybe part of the data are related to antiproliferative properties. For this reason, it is important to carry out approaches that reveal the role of senotherapy more precisely. Grosse et al., showed that cleared senescent endothelial cells (p16high-senescent cells) in the liver are not replaced adequately by non-senescent endothelial cells, resulting in increased vascular permeability in the liver and associated perivascular liver fibrosis [161]. Whether these effects also occur in PH is unknown. Therefore, combining genetic approaches such as the use of p16-ablator mouse models [161] to pharmacological approaches are needed to have a definitive interpretation of the role of cell senescence in the setting of PH.

Moreover, an important question is whether the loss of senescent cells caused by senotherapy may be detrimental to the progression of the disease. Endothelial and muscle cells loss combined with the limited regenerative potential of the vessels underlies the pathophysiology of PH. A recent study showed that counteracting cell senescence by navitoclax alters pulmonary hemodynamics in healthy animals and aggravates experimental PH [162]. This result contradicts those reported by Cullet et al. [119], which concluded that navitoclax reduces vascular remodeling and improves hemodynamics in in vivo animal models of PH. This controversy suggests that the role of senescent cells in the development of PH has not yet been elucidated and further study is needed.

Finally, we would like to mention that possibly depending on the PH subtype that is studied, the role of senescence may be different. In the PH associated with COPD or IPF, senescence may play a greater role because the age of the affected patients is usually older.

Thus, unlike diseases such as atherosclerosis or IPF where cellular senescence is well characterized, a conclusive effect of senolysis on PH development is still difficult. Therefore, the long-term effect of targeting senescent cells in the vasculature is largely unknown.

## 6. Conclusions

PH is a disease that affects the pulmonary vasculature, increasing pulmonary vascular resistance and pulmonary pressure, which leads to compensatory right ventricular hypertrophy, which can turn into right ventricular failure. The current approved therapies are limited, despite the improvement of quality of life that could be realized. They remain insufficient for reversing PH and improving survival. Every day, there is more evidence of the participation of senescence as a cellular process in the pathology of PH, although its role is still unclear. The data provided throughout the review provide a basis for further exploration of the role of senescence in PH and for clinical trials with senolytic drugs in PH patients.

## Figures and Tables

**Table 1:** Senescence markers.

Senescence Marker	Expected Change	Senescent Cell Hallmark
Telomere	Shortening of telomere length	Telomere shortening
β-galactosidase	Enzyme activity depends on pH (in senescent cells at pH6 and in normal cells at pH 4)	Increased lysosomal compartment and activity
Heterochromatin foci	Over-expression of heterochromatin proteins such as H3K9Me2/3, macroH2A, and HP1	Chromatin reorganization (SAHFs formation)
Histone γH2AX	Upregulated	DNA damage
53BPI	Overexpression	DNA damage
Bcl-2	Overexpression	Apoptosis resistance (DNA damage)
Lamin B1	Downexpression	Morphological changes (Nuclear membrane)
P53	Overexpression of p53	Cell cycle arrest (Activation of p53-p21 axis)
P21	Overexpression of p21	Cell cycle arrest (Activation of p53-p21 axis)
P16	Overexpression of p16INK4a	Cell cycle arrest (Activation of p16-pRB axis)
pRb	Overexpression of pRb	Cell cycle arrest (Activation of p16-pRB axis)
Ki67	Downregulation	Cell cycle arrest (Lack of proliferation)
EdU/BrdU	Lack of edU/BrdU incorporation	Cell cycle arrest (Lack of proliferation)
MMP2	Upregulated	Senescence-associated secretory phenotype
IL6	Upregulated	Senescence-associated secretory phenotype

**Table 2:** Clinical classification of pulmonary hypertension (PH). Adapted from [40].

**1. Pulmonary Arterial Hypertension**
1.1. Idiopathic 1.2. Heritable 1.2.1 BMPR2 mutation 1.2.2 Other mutations 1.3. Drugs and toxins induced 1.4. Associated with: 1.4.1. Connective tissue disease 1.4.2. Human immunodeficiency virus (HIV) infection 1.4.3. Portal hypertension 1.4.4. Congenital heart disease 1.4.5. Schistosomiasis 1.5 PAH long-term responders to calcium channel blockers 1.6 PAH with overt features of venous/capillaries (PVOD/PCH) involvement 1.7 Persistent PH of the newborn syndrome
**2. PH due to Left Heart Disease**
2.1. PH due to heart failure with preserved LVEF 2.2. PH due to heart failure with reduced LVEF 2.3. Valvular heart disease 2.4. Congenital/acquired cardiovascular conditions leading to post-capillary PH
**3. PH due to Lung Disease and/or Hypoxia**
3.1. Obstructive lung disease 3.2. Restrictive lung disease 3.3. Other lung disease with mixed restrictive/obstructive pattern 3.4. Hypoxia without lung disease 3.5 Developmental lung disorders
**4. PH due to Pulmonary Artery Obstructions**
4.1. Chronic thromboembolic PH 4.2. Other pulmonary arteries OBSTRUCTIONS 4.2.1. Sarcoma or angiosarcoma 4.2.2. Other malignant tumors Renal carcinoma Uterine carcinoma Germ cell tumors of the testis 4.2.3 Non-malignant tumors Uterine leiomyoma 4.2.3. Arteritis without connective tissue disease 4.2.4. Congenital pulmonary arteries stenosis 4.2.5. Parasites Hydatidosis
**5. PH with Unclear and/or Multifactorial Mechanisms**
5.1. Hematological disorders: chronic hemolytic anemia, myeloproliferative disorders 5.2. Systemic and metabolic disorders: sarcoidosis, pulmonary Langerhans cell histiocutosis, Gaucher disease, neurofibromatosis. 5.3. Others: chronic renal failure with or without hemodialysis, fibrosing mediastinitis. 5.4. Complex congenital heart disease

**Table 3:** Summary of senescence markers’ expression and distribution studies in animal models and cell types in PH.

Senescence Marker	PH, PH Animal Model and/or Cell Type	Senescence Marker Distribution	Senescence Marker mRNA and Protein Expression	Reference
P53	PAECs from HPH mice		Increased protein expression	[43]
	PAECs from rats with MCT		Increased protein expression	[43]
	PASMCs from HPH mice		Decreased protein expression	[43,44]
	PASMCs from rats with MCT		Decreased protein expression	[43]
	PASMCs and PAECs from patients with iPAH		Increased protein expression in iPAH-PAECs and reduced p53 expression in iPAH-PASMCs	[43,45]
P21	Mice with HPH and rats treated with MCT	Lung tissue, endothelial cells	Increased mRNA and protein expression in lung tissue	[41,47,49,50,51,52,53]
P16	Mice with HPH	Mainly located in the adventitia	Increased protein expression in older hypoxic mice	[41,49]
Bcl2	Mice with HPH	Pulmonary arterial walls	Increased protein expression	[58]
	PASMCs from HPH mice		Increased mRNA and protein expression	[58]
MMP2	Rats treated with MCT	Endothelium and adventitia and in right ventricle		[41,59]
IL-6	Rats treated with MCT	Adventitia and diffuse staining in media/neointima		[41]

Abbreviations: CHD: congenital heart disease; COPD: chronic obstructive pulmonary disease; HFpEF: heart failure with preserved ejection fraction; HPH: hypoxia-induced pulmonary hypertension; iPAH: idiopathic pulmonary arterial hypertension; MCT: monocrotaline; PAEC: pulmonary artery endothelial cell; PASMC: pulmonary artery smooth muscle cell.

**Table 4:** Summary of senescence markers’ expression and distribution studies in patients with PH.

Senescence Marker	Group of PH	Senescence Marker Distribution	Senescence Marker mRNA and Protein Expression	Reference
P21	PAH, CHD-PH and COPD-PH	Plexiform lesions		[41,54]
P16	Severe iPAH and COPD-PH	Plexiform lesions in pulmonary artery		[49,54,55]
Bcl2	HFpEF		Low values of endothelial Bcl2 index	[57]
	PAH		High values of endothelial Bcl2 index and Bcl2 in lung tissue	[56,57]
Survivin	PAH	Luminal cells of severe lesions		[41]
MMP2	iPAH		Increased protein expression in serum and urine	[60]
IL-6	iPAH		Increased protein expression in serum and lungs	[61,62]

Abbreviations: CHD: congenital heart disease; COPD: chronic obstructive pulmonary disease; HFpEF: heart failure with preserved ejection fraction; iPAH: idiopathic pulmonary arterial hypertension.
